# Sensor-Centric Intelligent Systems for Soybean Harvest Mechanization in Challenging Agro-Environments of China: A Review

**DOI:** 10.3390/s25216695

**Published:** 2025-11-02

**Authors:** Xinyang Gu, Zhong Tang, Bangzhui Wang

**Affiliations:** 1 School of Agricultural Engineering, Jiangsu University, Zhenjiang 212013, China; 2112316033@stmail.ujs.edu.cn (X.G.); 2222316058@stmail.ujs.edu.cn (B.W.); 2 Key Laboratory Equipment of Modern Agricultural Equipment and Technology, Jiangsu University, Ministry of Education, Zhenjiang 212013, China

**Keywords:** soybean, agricultural robot technology, agriculture in hilly and mountainous areas, multi-sensor fusion, terrain and crop sensing, precision agriculture

## Abstract

Soybean–corn intercropping in the hilly–mountainous regions of Southwest China poses unique challenges to mechanized harvesting because of complex topography and agronomic constraints. Addressing the soybean-harvesting bottleneck in these fields requires advanced sensing and perception rather than purely mechanical redesigns. Prior reviews emphasized flat-terrain machinery or single-crop systems, leaving a gap in sensor-centric solutions for intercropping on steep, irregular plots. This review analyzes how sensors enable the next generation of intelligent harvesters by linking field constraints to perception and control. We frame the core failures of conventional machines—instability, inconsistent cutting, and low efficiency—as perception problems driven by low pod height, severe slope effects, and header–row mismatches. From this perspective, we highlight five fronts: (1) terrain-profiling sensors integrated with adaptive headers; (2) IMUs and inclination sensors for chassis stability and traction on slopes; (3) multi-sensor fusion of LiDAR and machine vision with AI for crop identification, navigation, and obstacle avoidance; (4) vision and spectral sensing for selective harvesting and impurity pre-sorting; and (5) acoustic/vibration sensing for low-damage, high-efficiency threshing and cleaning. We conclude that compact, intelligent machinery powered by sensing, data fusion, and real-time control is essential, while acknowledging technological and socio-economic barriers to deployment. This review outlines a sensor-driven roadmap for sustainable, efficient soybean harvesting in challenging terrains.

## 1. Introduction

As a leading global source of plant protein and edible oil, soybeans (*Glycine max* L.) hold irreplaceable strategic importance for national food security and sustainable agricultural development [1,2]. Realizing this potential, however, is severely constrained by harvesting barriers in these challenging agro-environments, which directly threaten regional productivity and food security. Corn (*Zea mays* L.) is also China’s main food and feed crop [3]. This issue is not isolated to one country; similar mechanization barriers are found in the terraced fields of Southeast Asia and the Andean agricultural systems of South America, highlighting a problem of global significance. Hilly–mountainous areas in Southwest China, accounting for one-third of the nation’s total cultivated land, significantly influence China’s overall agricultural progress [4]. To quantify this challenge, the comprehensive mechanization rate for major crops in these regions is often below 40%, in stark contrast to the over 85% on the plains, with a significant portion of this land featuring slopes exceeding 15 degrees, where conventional machinery is ineffective. The terrain, particularly in the eastern Yunnan-Guizhou Plateau and Sichuan Basin, is complex, with scattered, scarce arable land often fragmented into slopes and terraces. Because these primary obstacles are fundamentally problems of perception and adaptation that exceed the capabilities of traditional mechanical designs, a sensor-centric review is critically needed to analyze the technological solutions required to overcome them. In this context, the soybean–corn intercropping system, an efficient composite agricultural model, is widely promoted across the southwest [5,6]. It boasts significant advantages: improving land-use efficiency, increasing the multiple cropping index, enhancing soil fertility, reducing pest incidence, and boosting overall crop yield potential [7,8]. Beyond alleviating land competition for grain and oil, this system leverages the southwest’s unique ecology. Corn offers natural support to soybeans, reducing lodging, while soybeans, through nitrogen fixation by root nodules, provide nutrients for corn [9,10]. This symbiotic relationship ensures complementary advantages and synergistic yield increases, significantly supporting regional agricultural development [11].

**Figure 1:**
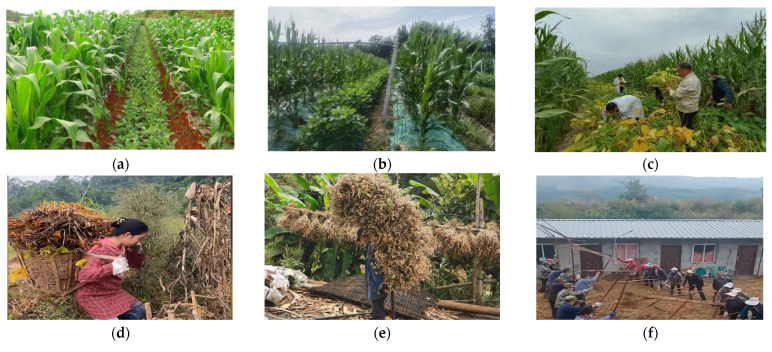
Soybean-harvesting process and current status in southwestern areas, illustrating key mechanization challenges. (**a**) Intercropped soybean and corn on hilly terrain, requiring precise navigation and selective harvesting technologies; (**b**) hilly terrain for cultivation, limiting the size and stability of conventional machinery; (**c**) manual pulling and harvesting of soybeans, representing the low-efficiency baseline that mechanization aims to replace; (**d**) manual carrying of soybeans, highlighting the logistical challenges in fragmented terrain; (**e**) sun-drying of harvested soybeans, an inefficient post-harvest step that combines are designed to eliminate; (**f**) manual soybean threshing, a process prone to high grain loss and damage, which mechanical systems must improve upon.

However, as highlighted in Figure 1, soybean harvesting remains the critical bottleneck in Southwest China. Small, fragmented fields on sloping terrain, irregular row patterns, and frequent lodging make conventional combines impractical, while hand cutting, portable reapers, and standalone threshers still dominate [12,13]. This mechanization gap is fundamentally a perception and adaptation problem, where conventional machines lack the sensory feedback to handle variability. Compared to China’s northern plains, where soybean production is largely mechanized, harvesting in Southwest China’s hilly–mountainous regions still heavily relies on traditional manual methods [14]. These methods are time-consuming, labor-intensive, inefficient, and costly, leading to high losses. This labor-intensive approach results in extremely low production efficiency, escalating labor costs, and persistently high harvest losses. It directly discourages farmers from planting soybeans and severely impedes the regional industry’s modernization and intensification [15,16].

This paper aims to comprehensively map and deeply analyze the bottlenecks in soybean harvest mechanization within the soybean–corn intercropping pattern in Southwest China’s hilly–mountainous areas. Specifically, the objectives of this review are the following: (1) identify the key sensor-based technological frontiers, (2) analyze the limitations of current systems from a perception standpoint, and (3) propose a technological roadmap for developing the next generation of intelligent harvesters. Specifically, this review focuses on five core areas: (1) The impact of agronomic features and severe terrain on mechanization adaptability, which define the core perception requirements for intelligent machinery; (2) grain loss and plant damage patterns of existing equipment, and the role of monitoring sensors in their quantification; (3) crop characteristic research, equipment development, and the application of emerging technologies, with a focus on advanced sensing systems (e.g., LiDAR and machine vision); (4) sensor-based precise identification, selective harvesting, and pre-separation of impurities for intercropped soybeans; and (5) low-damage, high-efficiency threshing and optimized cleaning performance for hilly slope operations, as guided by real-time process sensors. By thoroughly dissecting and summarizing these critical issues and current research progress, this paper seeks to provide the theoretical basis and practical guidance for overcoming the mechanization bottleneck in soybean–corn intercropping in Southwest China. Ultimately, it aims to promote agricultural modernization and sustainable development in the region. Our research began with a comprehensive literature review. Relevant articles were retrieved from the Web of Science database and “China National Knowledge Infrastructure” (CNKI), with a total of 214 manuscripts reviewed.

## 2. Materials and Methods

We initiated the study by conducting a comprehensive literature review. Articles were retrieved from the Web of Science database and “CNKI” (China National Knowledge Infrastructure), resulting in the examination of 214 manuscripts.

Search Strategy (for databases like Web of Science and CNKI):

(“soybean harvesting” OR “soybean harvest mechanization”) AND (sensor* OR “intelligent system*” OR “agricultural robot”) AND (“hilly” OR “mountainous” OR “challenging terrain” OR “complex terrain”); (“soybean-corn intercropping” OR “maize-soybean intercropping”) AND (“harvest*” OR “mechanization”) AND (“robot*” OR “sensor fusion” OR “machine vision” OR “LiDAR”); (“precision agriculture” OR “smart farming”) AND “soybean harvest*” AND (“sensor technology” OR “IMU” OR “GPS”); (“agricultural machinery” OR “combine harvester”) AND “terrain sensing” AND “soybean”; Review AND “soybean harvesting” AND “intelligent agriculture”

The Inclusion criteria were as follows:

(1) publication type must be a peer-reviewed journal article, a high-level international conference paper, or a doctoral dissertation; (2) research content must directly focus on sensor technologies, intelligent control systems, robotics, or related mechanized equipment applied to the soybean-harvesting process; (3) the research context must be explicitly set in hilly and mountainous areas, intercropping systems, or other complex agricultural environments with similar challenges; and (4) the language of publication must be English or Chinese.

Correspondingly, the exclusion criteria were as follows: (1) non-academic publications, such as patent specifications, news articles, product manuals, technical white papers, and non-peer-reviewed online resources; (2) studies with irrelevant topics, for instance, those focusing solely on soybean genetics, agronomy, pest control, or food processing, without substantively addressing the mechanization and intelligent technologies of the harvesting stage; (3) previously published review articles, to avoid information redundancy and circular reasoning; and (4) duplicate publications of the same research (e.g., both conference and journal versions), in which case only the most comprehensive version was retained.

## 3. Agronomic Characteristics and Severe Terrain Constraints of Soybean–Corn Intercropping Pattern in Southwest Hilly–Mountainous Areas

The unique topography and intercropping agronomic features of Southwest China’s hilly–mountainous regions are fundamental constraints on soybean harvest mechanization. These constraints are distinct from those in other regions, which may face large-scale logistics but not the simultaneous challenge of steep slopes combined with complex, multi-crop agronomy. These factors directly impact harvester adaptability, operational efficiency, and harvest quality. For example, poor adaptability to terrain directly calls for advanced terrain-profiling sensors, while low operational efficiency in intercropped rows necessitates robust machine vision for precise navigation.

### 3.1. Agronomic Characteristics of Soybean–Corn Intercropping Patterns in Southwest China’s Hilly–Mountainous Areas

**Figure 2:**
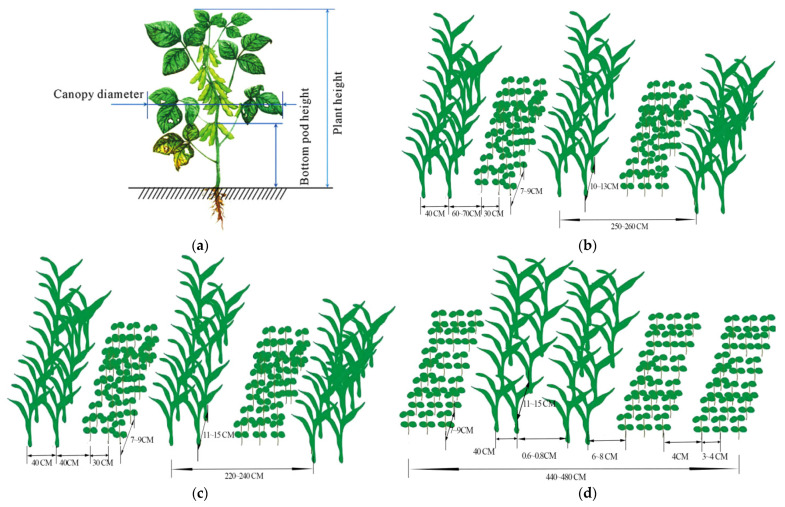
Soybean biological characteristics and planting patterns in Southwest China. (**a**) Soybean plant morphology parameter measurement; (**b**) 2 rows of corn and 4 rows of soybean strip intercropping pattern; (**c**) 2 rows of corn and 3 rows of soybean strip intercropping pattern; (**d**) 2 rows of corn and 6 rows of soybean strip intercropping pattern.

Widely cultivated soybean varieties in Southwest China’s hilly–mountainous areas are typically high-yielding local adaptations [17]. However, these varieties often exhibit low podding heights, typically 5–10 cm, which is significantly below the 15 cm required for mechanized harvesting on plains. This single factor can contribute to harvest losses often exceeding 10–15% before the crop even enters the machine. This directly contributes to high soybean harvest losses in the southwest [18]. Furthermore, when intercropped with tall corn, soybean plants experience light competition and restricted growth space, leading to irregular plant architecture: weak stems, numerous branches, and poor lodging resistance. Upon maturity, soybeans are highly prone to lodging and entanglement, complicating row alignment and increasing clogging risks during mechanical harvesting [19,20]. Such unpredictable plant postures cannot be managed by fixed mechanical systems, directly demonstrating the need for advanced sensing systems, like machine vision or LiDAR, to dynamically adapt the header and reel in real-time. Soybean–corn intercropping patterns in Southwest China’s hilly–mountainous areas are diverse, commonly including 3:2, 4:2, and 6:4 ratios. The spacing between the two crops is typically maintained at 60–70 cm [21], as shown in Figure 2. Studies suggest that optimal row spacing for corn and soybeans is 4 cm and 30 cm, respectively, though farmers often adjust these distances based on their machinery and specific terrain.

**Figure 3:**
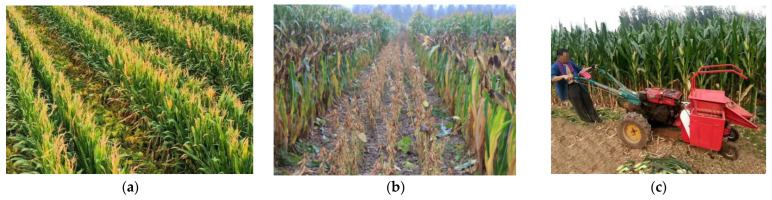
Different harvesting methods/practices for soybean–maize intercropping. (**a**) Soybeans mature first in intercropping pattern; (**b**) unified harvest after corn matures; (**c**) corn harvested after soybeans.

However, the cutter widths of general-purpose harvesters, usually ranging from 1.8 to 3 m, are poorly matched to the varied intercropping strip patterns in the southwest. This mismatch makes it difficult for machines to accurately align with crop rows during harvest, frequently leading to damage to corn plants and missed or crushed soybeans [22]. In practice, such header- and row-spacing mismatches have been documented to increase soybean-harvesting losses by an additional 5% to 8% at the header alone. In intercropping pattern, as shown in Figure 3, soybeans mature 15 to 30 days earlier than corn. This means that when soybeans are ready for harvest, corn is largely still in its grain-filling stage. A single-pass harvesting approach would inevitably lead to corn loss. However, staged harvesting, while protecting crops, significantly increases machine operation frequency, labor input, and operational costs, thereby reducing the economic feasibility of mechanization in the southwest [23].

In summary, the agronomic characteristics of soybean–corn intercropping in Southwest China’s hilly–mountainous areas pose three main challenges for mechanized harvesting, each demanding specific sensing and perception capabilities: (1) The challenging biological characteristics of soybean varieties, such as low pod height and lodging, which require advanced terrain- and crop-profiling sensors for adaptive harvesting; (2) the diversity and complexity of intercropping patterns, which necessitate robust machine vision and navigation sensor systems for real-time row identification and precise guidance; (3) the asynchronous maturity periods of the two crops, demanding sensor-based perception systems capable of distinguishing and selectively harvesting only the mature soybean plants.

### 3.2. Significance of Soybean Cultivation in Southwest China for Mechanized Harvesting Development

**Figure 4:**
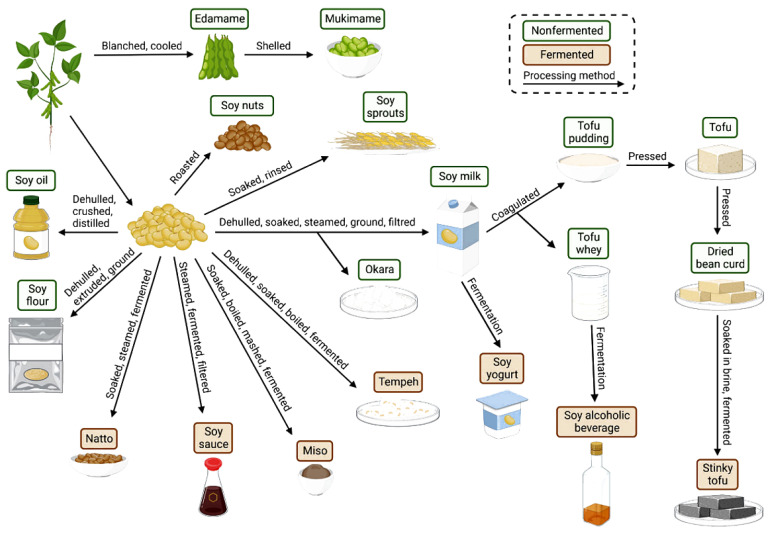
Diagram of soybean resource utilization and product development: Integrating fermentation and non-fermentation technologies in the food industry.

Soybeans, known as “meat grown from the earth, ” hold a pivotal place in nutrition. They are one of the few plant-based foods that provide complete protein [24], containing all essential amino acids, making them an ideal choice for vegetarians and those seeking diverse protein sources [25] (Figure 4). Additionally, soybeans are rich in heart-healthy unsaturated fatty acids and are cholesterol-free. Their abundant dietary fiber aids gut health and blood sugar stability [26]. They are also a treasure trove of minerals like calcium, iron, and potassium, alongside unique plant bioactives such as soy isoflavones and lecithin. These components play vital roles in antioxidation, bone health, and regulating physiological functions [27]. Thus, with their comprehensive and exceptional nutritional value, soybeans provide a solid food foundation for balanced diets, chronic disease prevention, and overall health, and they can also be processed into products like soy sauce [28,29].

Promoting soybean cultivation in Southwest China offers multiple benefits, serving as a systemic project integrating ecological protection, economic development, and social well-being [30]. Ecologically, soybean root nodules fix nitrogen, enriching soil and reducing fertilizer use; the “corn-soybean strip intercropping” pattern achieves “double harvests from one field” while effectively conserving soil and water and mitigating rocky desertification [31]. Economically, soybeans directly boost farmer income and supply raw materials for processing industries like Pixian Doubanjiang, forming a “planting-processing-sales” industrial chain that drives rural revitalization [32]. Given China’s high reliance on soybean imports, this initiative is also crucial for improving dietary nutrition and ensuring national grain and oil security [33].

Currently, soybean harvesting in Southwest China heavily relies on manual labor, resulting in high costs and low efficiency. Large combine harvesters designed for flat plains are not fully adapted to the complex terrain and intercropping patterns of the southwest [34,35]. Furthermore, soybean plants inherently exhibit biological characteristics such as low podding height, susceptibility to pod shattering, and lodging during harvest. Direct operation by existing machinery easily leads to grain breakage and scattering losses, resulting in high overall loss rates.

This is precisely where the significance of sensor research becomes paramount. A critical research gap, therefore, exists in the development of robust sensor fusion algorithms that can reliably distinguish between intercropped soybeans and corn under variable field conditions. These challenges highlight that the core limitation of current machinery is its inability to sense and adapt to variability. Advanced sensor research aims to provide machines with the “eyes” and “feel” they currently lack: terrain-profiling sensors to capture low-lying pods, machine vision to navigate complex intercropping patterns and detect lodged plants, and vibration or acoustic sensors to adjust operations in real-time to minimize pod shattering.

## 4. Severe Constraints and Their Impact from Hilly–Mountainous Terrain

Addressing the agronomic characteristics and harvesting challenges of various crops in hilly–mountainous areas, numerous scholars have researched harvesting equipment. Zhao Zhu et al. [36] and Geng, Duanyang et al. [37] designed corn harvesters capable of stable slope operation using TRIZ theory or orthogonal experimental design. Wang Shuguang et al. [38] optimized corn ear-picking devices via ADAMS simulation and response surface methodology to reduce loss rates. Li Ping et al. [39], Kong, Chunyan et al. [40], and Sun Xiaoxiao et al. [41] used the discrete element method (EDEM) or gas–solid coupling simulations to optimize the terrain adaptability of soybean threshing, potato-digging shovels, and rapeseed-cleaning devices, respectively. Additionally, researchers successfully developed specialized equipment for complex regions, such as sweet potato vine-removing and digging-integrated machines [42], self-propelled medicinal plant excavators [43], camellia fruit vibration harvesters [44], and small peanut combine harvesters [45], through integrated design methods or field trials. Yi, Fengyan et al. [46], Zhang, Danzhu et al. [47], and Shi, Meiqi et al. [48] designed adaptive cutting table height or canopy-profiling harvest systems for corn, tea, and sugarcane harvesters using adaptive control algorithms or LiDAR technology. Yu, Yongchao et al. [49] developed an automatically leveling high-altitude work platform for hilly orchards based on incremental PID control. While these studies represent significant progress, they often focus on optimizing individual components in isolation, seldom addressing the holistic challenge of integrating these subsystems into a single, dynamically adaptive machine.

**Figure 5:**
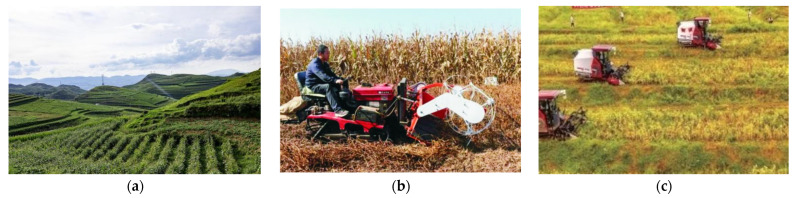
Analysis of mechanization challenges for soybeans in hilly and mountainous regions. (**a**) Steep slopes, uneven ground, and operational safety hazards; (**b**) small soybean harvester operating in hilly–mountainous areas; (**c**) soybean combine harvester only suitable for relatively flat terrain, with cross-slope and longitudinal slope ≤ 10°.

In conclusion, as shown in Figure 5, the primary terrain constraints facing existing harvesting equipment in Southwest China’s hilly–mountainous regions can be recast as perception-centric problems that require advanced sensor fusion:

(1) Small, irregular, and fragmented plots [50]. Limited operational space and heterogeneous boundaries depress efficiency. Mitigation demands more than a single positioning sensor: fuse RTK-GNSS with an IMU for continuous, robust localization, and integrate LiDAR or machine vision to delineate irregular plot edges and enable effective path planning.

(2) Steep slopes, uneven ground, and safety risks [51]. Rugged terrain prevents stable ground contact and degrades control. Fusing IMU-based attitude with terrain-profiling sensors allows the system to distinguish machine tilt from ground undulation, supporting coordinated chassis-leveling and header height control to avoid “high cutting” or “digging into the soil” [52].

(3) Dense ridges, ditches, and difficult transfers [53]. Access and transfer efficiency are severely constrained. Reliable detection exceeds the capability of any single modality; fusing LiDAR (for precise 3D structure) with machine vision (for semantic classification) enables accurate identification and navigation around ditches, ridges, and stone embankments, improving both safety and efficiency.

## 5. Grain Loss and Plant Damage Patterns of Existing Harvesting Machinery Operating in Complex Terrain and Intercropping

**Figure 6:**
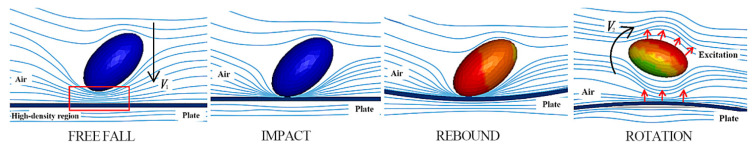
The detection method for cleaning loss based on acoustic signals.

Analyzing crop loss patterns is fundamental for harvesting machinery design and optimization, providing a basis for optimizing mechanical parameters. Li, Yang et al. [54] studied the impact of header vibration on rapeseed harvesting losses; an improved header reduced total vibration by 19.9% to 43.9% and total rapeseed header loss by 33.2% to 46.9%. Jin, Mingzhi and Liang, Zhenwei et al. [55,56] proposed a method to reduce combine harvester cleaning losses based on piezoelectric effect quantification. Xu, Lizhang et al. [57] developed a rapeseed loss-monitoring system based on impact signal analysis. Shen, Yuhao et al. [58] introduced a new sound signal-based detection method using the ALE algorithm to detect cleaning losses during combine harvester operation (Figure 6). Chen, J et al. [59] proposed adjusting machine parameters using knowledge discovery in databases (KDD) to reduce crop losses. Li, Yaoming et al. [60] found that moisture content and loading rate influence crop breakage and loss characteristics.

**Figure 7:**
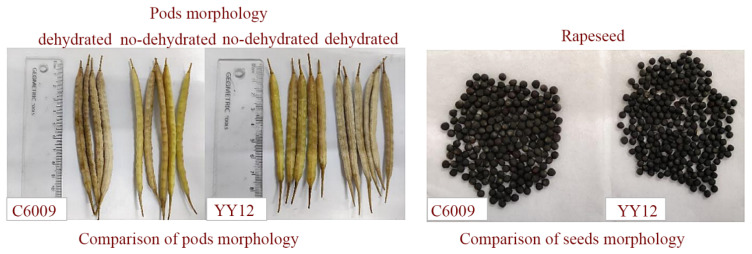
The influence of moisture content and loading rate on crop breakage and loss characteristics.

Chen, Jin et al. [61] designed a sampling device and proposed a machine vision-based real-time monitoring method for rice combine harvester breakage rates. Chandio et al. [62] found that corn grain characteristics are related to the design of planters, harvesters, threshers, and processing machines. Qing, Yiren et al. [63,64] attributed significant rapeseed losses during mechanized harvesting primarily to a mismatch between harvester requirements and the crop’s pod shatter resistance and branching characteristics (Figure 7). Han, Dianlei et al. [65] used EDEM 2022 discrete element simulation software to establish a discrete element model for podded peppers, simulating ground drop losses during harvesting. Evidently, the primary losses and damages during crop harvesting are mainly categorized into grain loss and plant damage.

The research cited above highlights a clear and crucial trend: the move towards integrating dedicated sensor systems to quantify and understand crop loss mechanisms. The application of piezoelectric, acoustic, and machine vision sensors represents a significant leap from traditional, offline assessment methods. However, these technologies often serve as reactive monitoring tools—measuring loss after it has already occurred. The future of intelligent harvesting machinery design lies in a proactive, sensor-driven control paradigm. For instance, field trials have already demonstrated prototypes that use machine vision to predict incoming crop volume to proactively adjust forward speed and in-line acoustic sensors to dynamically regulate threshing intensity based on real-time grain impact. This involves creating a comprehensive perception system that not only detects loss but also senses the precursor conditions, such as crop moisture content, plant density, and header vibration. By fusing this data, an intelligent harvester could dynamically adjust its operational parameters—such as forward speed, reel rotation, and threshing intensity—in real-time to prevent losses before they happen, rather than simply measuring them.

### 5.1. Characterization of Soybean Harvest Losses in Hilly and Mountainous Areas

**Figure 8:**
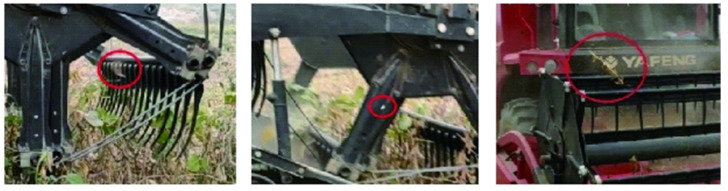
Header mismatch leading to stubble loss and shattering loss during soybean harvest.

The deep understanding of crop loss patterns during harvesting is crucial for improving and optimizing mechanical components, reducing operational energy consumption, and enhancing harvesting efficiency [66]. Specifically, this understanding is now enabled by deploying targeted sensing technologies, such as piezoelectric impact plates, to quantify grain loss from the cleaning shoe and machine vision systems to monitor grain breakage and impurity levels. Based on previous research into harvesting damage and losses, the primary loss patterns in soybean harvesting include the following (Figure 8) [67].

Stubble Loss: Due to the low podding height characteristic of soybean varieties in Southwest China’s hilly–mountainous regions, many pods are positioned below the minimum cutting height of general-purpose headers, leading to significant low-lying pod loss in the field [68]. When soybean plants lodge, or grow prostrate, or stems become entangled, the cutter bar cannot effectively cut them, drastically increasing stubble loss. Furthermore, the challenging terrain exacerbates inconsistencies in cutting height, resulting in excessively high stubble in some areas, which can account for 60–70% of total losses in certain cases [69].

Header Shattering/Vibration Loss: During harvest, the reciprocating motion of the cutter bar, the rotation of the reel, and the auger of the header generate significant vibrations [70]. These vibrations impact mature pods or grains, causing grains to detach from pods and scatter in the field [71,72]. This loss is particularly severe when soybean grains are highly mature and pods are dry and prone to shattering. This is a critical area where on-header acoustic or vibration sensors could be deployed to monitor vibration intensity in real-time and provide feedback to actively regulate reel speed, thus mitigating shattering losses.

Feeding Loss and Uncut Loss: As the header feeds crops into the threshing drum, mismatched feeding speed or poorly designed feeding mechanisms can cause some pods to detach or be thrown out during the process [73]. Concurrently, imprecise row alignment or incompatible cutting width leads to some soybean plants not being effectively harvested; instead, they are directly crushed by the machinery and left in the field, resulting in uncut loss [74].

Collectively, these loss patterns highlight a critical deficiency in conventional harvesting machinery: the lack of a comprehensive real-time perception system. Stubble loss and uncut loss are direct consequences of inadequate terrain-profiling and navigation sensor systems that fail to precisely adapt the header’s position to the ground and crop rows. Concurrently, shattering and feeding losses stem from the absence of process sensors (e.g., for vibration, torque, or crop flow) that could provide the feedback needed to dynamically regulate operational intensity.

### 5.2. Impact of Plant Damage Patterns on Intercropping Crops

**Figure 9:**
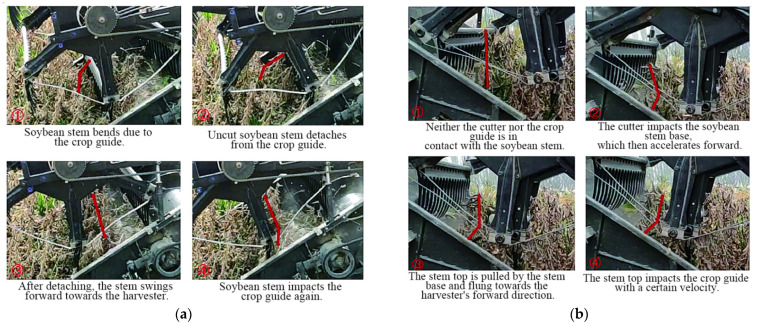
Movement characteristics of the soybean crop during harvesting. (**a**) Stem movement when the reel’s operating range is small; (**b**) interaction characteristics between header and soybean stems when the machine is mismatched.

Analyzing crop damage patterns is fundamental for harvesting machinery design and optimization. A deep understanding of crop damage patterns during harvest is crucial for improving and optimizing mechanical components, reducing operational energy consumption, and enhancing harvesting efficiency. Based on previous research into harvesting damage and losses, the primary loss patterns in the intercropping pattern include the following (Figure 9) [75]:

(1) Soybean Plant Damage: The rigid structure of general-purpose headers often causes excessive pulling and crushing when harvesting low-lying, lodged soybeans. This is a direct result of the machine’s inability to sense plant posture and dynamically adapt its header height and reel speed. This mechanical insensitivity leads to pod shattering and stem breakage, resulting in grain loss. Plants that cannot be lifted are directly missed—a failure of the visual or profiling sensor system—and mechanical impact can also cause latent damage to grains, reducing market quality. For example, research has shown that excessive mechanical impact can increase the rate of internal micro-cracks in soybean seeds by over 5%, which compromises both seed viability for planting and their grade for food processing [76].

(2) Accidental Corn Plant Damage: In strip intercropping, the lack of a robust perception system—typically based on machine vision or LiDAR—for precise crop-row identification means general-purpose harvesters cannot accurately align with the target soybean rows [77]. This sensory deficit often leads to accidental damage or crushing of adjacent corn plants, directly impacting corn yield and quality. Furthermore, without a sensor system to detect blockages, severed corn stalks can entangle mechanical components, reducing operational efficiency.

(3) Soil Environmental Damage: When large harvesters operate in hilly–mountainous areas, their significant weight severely compacts the soil. This is exacerbated by non-optimal path planning that could be improved by leveraging RTK-GNSS positioning data and digital elevation models [78]. Concurrently, frequent turning and braking on slopes significantly exacerbate the risk of soil erosion, particularly when the soil is wet—a condition that could be monitored by on-board or remote soil moisture sensors to inform safer operational decisions.

## 6. Key Analysis of Soybean-Harvesting Equipment Technology Research for Complex Agronomy and Rugged Terrain

### 6.1. Research Status of Interacting Mechanical Property Factors During Mechanical Harvesting

**Figure 10:**
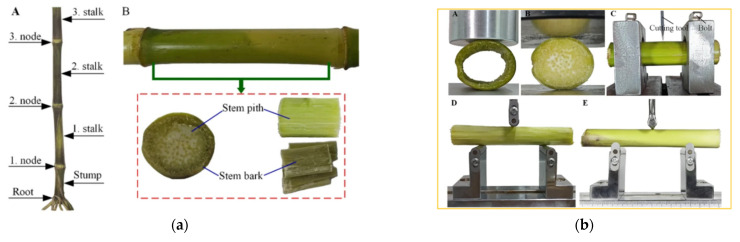
Research on crop stems/stalks to advance the development of mechanized harvesting. (**a**) Study on crop stem component properties. (A) dividing of the cornstalk, (B) structural compositions of the inter-nodal section of the cornstalk.; (**b**) schematic of mechanical performance test for maize stalks and their components. (A) stem bark compression test, (B) cornstalk compression test, (C) cornstalk shear test, (D) stem pith bending test, (E) cornstalk bending test.

The deep understanding of the complexity of mechanical behavior and its relationship with plant physiology and environmental adaptation is crucial for revealing sources of resistance during mechanical operation, enhancing harvesting efficiency, and optimizing operational parameters. Gao, Qimin et al. [79] and Wang, Wei et al. [80] used universal material-testing machines to study the shear and bending properties of tomato and millet stems, providing foundational data for harvester optimization (Figure 10). Peng, Zehui et al. and Zhao, Yunfei et al. [81,82] conducted comprehensive mechanical tests on chili and broccoli stems, providing key parameters for mechanical modeling of transplanting and cutting equipment. Hidalgo-Cordero, Juan Fernando et al. and Wojtowicz, Tomasz et al. [83,84] evaluated the mechanical performance of reed and rye stems to study crop production performance. Han, Dandan et al. [85] performed physical tests on corn stalks and developed a discrete element model (DEM) to simulate and predict mechanical behavior during harvesting (Figure 10).

**Figure 11:**
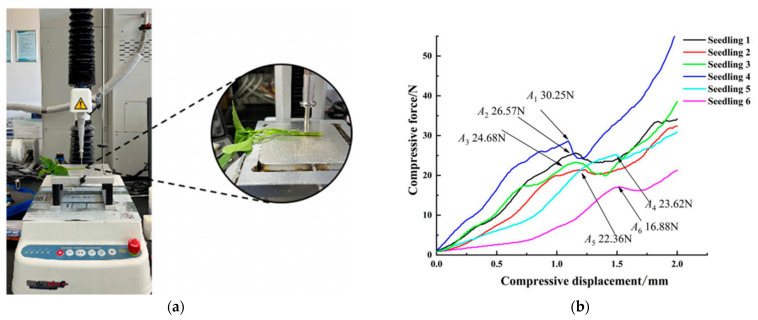
Methodology for obtaining the mechanical behavior curves of crop stems. (**a**) Stem compression characteristic test; (**b**) study on stem displacement characteristics.

Gianinettiet et al. [86] precisely identified the mechanical weak points (nodes) of barley stems using three-point bending tests, revealing the significant impact of humidity on stem-bending strength. Zargar et al. [87] employed creep tests to study the viscoelasticity of sorghum stems, which is vital for understanding lodging mechanisms under long-term loads. Kaminski et al. [88] utilized four-point bending and torsion tests to comprehensively analyze the spatiotemporal dynamic changes in plant-bending and torsional mechanical properties throughout their development cycle. Wu, Wenchao et al. [89] investigated the bending behavior of tea stems under large deflection conditions, which is applicable to the optimized design of tea-picking machine cutters and adaptive parameter adjustment (Figure 11). Wu, Shu et al. [90] studied the interaction characteristics between canopy openers and rice straw based on transient dynamics. Li, Ziyu et al. [91] researched the influence of cutting parameters on the ultimate shear stress and specific cutting energy of daylily stems.

For soybeans specifically, experimental data show that stem shear strength, which varies with moisture content, is a critical parameter for optimizing cutterbar knife speed and geometry to minimize cutting losses and power consumption. Analyzing stem shear and bending characteristics is fundamental for optimizing cutting devices and reducing loss and consumption [92]. Combining this with numerical simulations allows for predicting plant-lodging behavior on slopes, thereby enabling equipment optimization. Furthermore, investigating humidity and viscoelasticity helps reveal lodging mechanisms, which can then determine the optimal harvest timing and the adhesion characteristics of harvesting equipment. Ultimately, a comprehensive understanding of the mechanical dynamics throughout the crop’s growth cycle ensures precise matching of equipment parameters to plant status, achieving efficient, low-loss harvesting.

The research on stem mechanical properties offers an essential engineering basis for harvesting component design. However, these properties are field-variable—shifting with moisture content, growth stage, and lodging severity. This underscores the role of on-board sensing. Rather than relying on fixed parameters derived from laboratory averages, an intelligent harvester should exploit real-time signals—e.g., machine vision for lodging assessment, force/torque sensing at the cutter for cutting resistance, and near-infrared (NIR) for biomass moisture—to infer the current mechanical state of the crop. Fusing this foundational knowledge with live sensor feedback enables a shift from static optimization to dynamic adaptation, allowing the machine to adjust settings in real-time for truly efficient, low-loss harvesting.

### 6.2. Existing Harvesting Equipment and Research Methods in Southwest China

Addressing the complex topography and intercropping agronomy of Southwest China’s hilly–mountainous areas necessitates developing harvesters with superior ground profiling, operational stability, and intelligent control. Many scholars have researched the equipment–terrain mismatch: Wang, Shiguo et al. [77], Jin Chengqian et al. [93], and Ni, Y. et al. [94] explored header ground profiling and adaptive height adjustment using mechanical design, sensor detection, and MBD-DEM coupled simulation. Field trials of these intelligent headers have demonstrated significant performance gains, with some active profiling systems reducing stubble losses by up to 50% compared to conventional rigid headers in uneven terrain. Leng, Yuancai et al. [95] investigated adaptive reel parameter adjustment via mechatronic control and multi-body dynamics simulation. Zhou Weiwei et al. [96] and Li Zerui et al. [97] designed soybean headers with terrain fluctuation detection for real-time height adjustment. Liu Gangwei [98] and Huang Chunyang [99] designed header height adjustment systems integrating height detection, information processing, and adjustment. Xie Hongru [100] and Li Zerui [101] specifically designed harvester headers based on hilly terrain features and soybean plant physiology.

**Figure 12:**
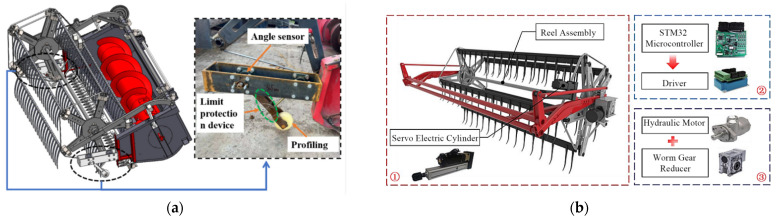
Design of a soybean header for complex terrain. (**a**) Flexible header and active ground-profiling technology; (**b**) design of terrain-detecting adaptive profiling header.

**Figure 13:**
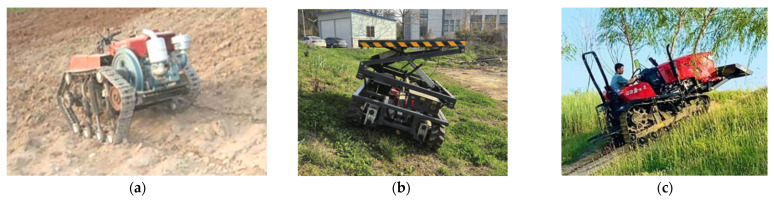

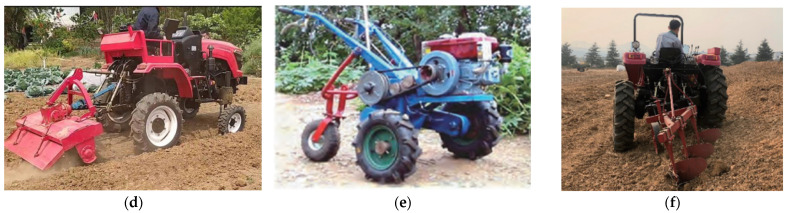
Design of a soybean header for complex terrain. (**a**) Contour-parallel driving of hilly tracked tractor with leveling; (**b**) chassis adjustment using neural network algorithm; (**c**) agile tractor for hilly–mountainous areas; (**d**) four-wheel independent leveling tractor technology and multi-functional walking system; (**e**) small walk-behind adaptive tractor for hilly–mountainous regions; (**f**) multi-functional walking system and traction application.

In summary, the primary intelligent stability control technologies for hilly regions include the following:

(1) Flexible headers with active ground profiling: This is a core optimization for uneven plots [102,103]. The system relies on hydraulic/mechanical linkages that are actively controlled by a suite of profiling sensors, such as ultrasonic sensors, LiDAR, or mechanical probes. These sensors provide high-resolution, real-time ground height data, enabling a closed-loop control system to dynamically adjust the cutter bar for precise “ground-hugging harvesting”, ensuring that low-lying pods are captured without scooping soil [104,105] (Figure 12).

(2) Whole machine attitude adaptive leveling is enabled by sensor fusion: Operating on steep, hilly–mountainous terrain, a tilted machine body severely impacts performance [106,107]. Intelligent leveling systems are a crucial technology built upon the principle of multi-sensor fusion. By integrating data from an inertial measurement unit (IMU)—which includes accelerometers and gyroscopes—with real-time kinematic global navigation satellite systems (RTK-GNSS), the system can precisely perceive the machine’s 3D attitude (roll, pitch) and absolute position in real-time. This fusion is critical, as the IMU provides high-frequency stability data while the RTK-GNSS corrects for long-term drift [108,109]. Based on this fused data, high-response actuators dynamically adjust the entire machine, ensuring optimal operational conditions for internal components like the threshing drum and cleaning sieve, as shown in Figure 13 [110].

(3) Sensor-enhanced walking system and traction optimization: Addressing the complex conditions of hilly–mountainous slopes, primary development directions include tracked or four-wheel-drive systems. While these mechanical solutions are foundational, their performance and safety are significantly enhanced through sensor-based control systems. Integrating wheel-speed sensors allows for real-time slip detection and intelligent traction control, automatically engaging differential locks or modulating power. Prototypes of such sensor-enhanced systems, particularly in specialized vineyard and forestry tractors, have shown the ability to reduce wheel slip by over 30% on steep slopes, validating the concept for challenging agricultural terrains. Furthermore, data from the IMU can be used to monitor dynamic stability, providing early warnings for potential rollovers. Future exploration includes forward-looking sensors like LiDAR or cameras to proactively assess terrain conditions (e.g., moisture and roughness) and adjust the drive control strategy before encountering hazardous terrains [111,112].

In essence, these three technological directions—from component-level ground adaptation to whole-machine attitude stabilization, and, finally, to dynamic traction control—collectively illustrate the evolution towards a fully sensor-aware harvesting platform. They underscore that the future of mechanization in complex terrain is not about perfecting isolated mechanical systems, but about their holistic integration through a centralized perception and control architecture. The ultimate goal is to fuse the data from profiling sensors, IMUs, GNSS, and wheel-speed sensors into a single, unified model of situational awareness. This enables a machine that does not just react to its environment in parts, but proactively and synergistically coordinates all its functions for maximum efficiency, safety, and performance.

### 6.3. Intelligent Navigation and Path Planning Technology

**Figure 14:**
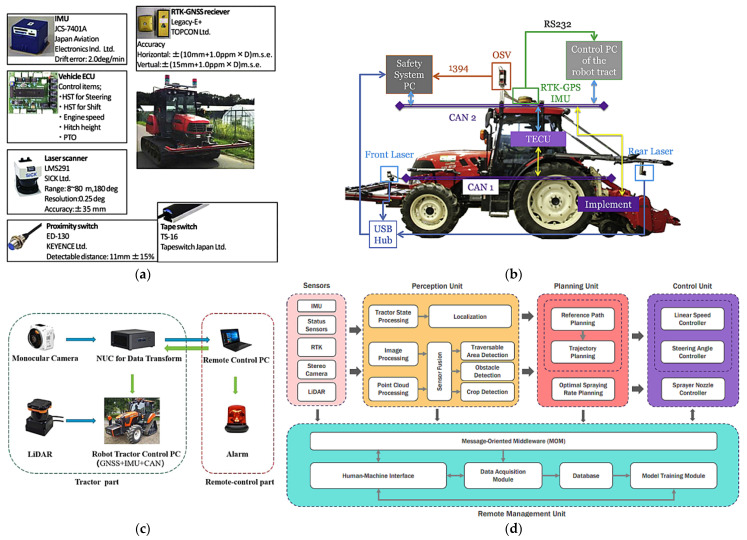
Research on an intelligent tractor suitable for operating harvesting equipment in hilly and mountainous areas. (**a**) Hardware of a robotic tractor for autonomous path planning and obstacle avoidance; (**b**) schematic diagram of the autonomous harvesting planning system for a robotic tractor; (**c**) remote safety system for a robotic tractor using a monocular camera and the YOLO vision-based obstacle-avoidance method; (**d**) overall architecture of the autonomous operation control system for intelligent agricultural machinery.

Intelligent navigation and path planning are critical research priorities for hilly–mountainous harvesting equipment. Zheng, Siqi et al. [113] achieved autonomous navigation and precise obstacle avoidance using a stereo vision-based 3D obstacle detection system and a Frenet optimal trajectory (FOT) planning algorithm, as shown in Figure 14.

Han, Jiangyi et al. [114] developed nonlinear obstacle-avoidance path planning for trailer systems via kinematic analysis and an adaptive homotopy algorithm. Guo, Chaoke et al. [115] designed a lateral path planner for autonomous tractor-trailer vehicles (ATTVs) using model predictive control (MPC) in the Frenet frame, suitable for narrow, structured roads. Jia, Bobo et al. [116] investigated trajectory planning for trailer systems in static and dynamic environments, employing nonlinear MPC (NMPC) combined with the RRT algorithm. Hellander, Anja et al. [117,118] proposed a combined task-and-motion planner based on heuristic-guided graph search to achieve efficient and near-optimal path integration for single and multiple tractors. Kolahi et al. [119] studied optimal point-to-point path planning for trailer-type mobile robots, incorporating wheel inertia through nonlinear modeling and Pontryagin’s minimum principle. Zhang, Jing [120] enhanced field navigation for agricultural machinery by fusing INS/GNSS data with an adaptive Kalman filter.

**Figure 15:**
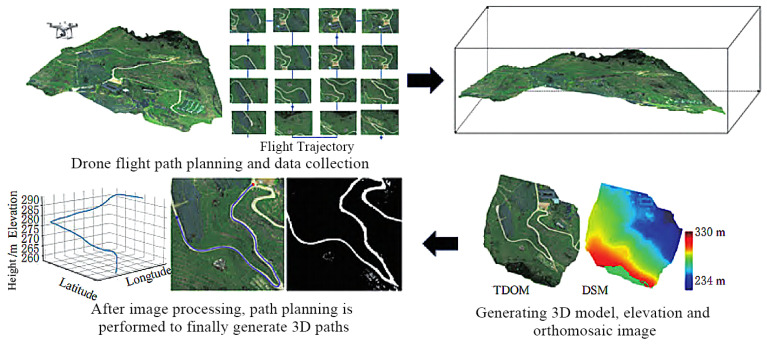
The 3D path-planning method flow for hilly–mountainous areas.

Research on navigation and obstacle avoidance in specific complex environments is crucial. Chang, Ningjie et al. [121] achieved autonomous obstacle avoidance for mountain tractors by constructing semantic 3D maps using semantic neural networks and laser SLAM, combined with the A* algorithm. Xu, Ning et al. [122] proposed a hybrid algorithm, combining improved bidirectional A* and AGADE, for global path planning of articulated steering tractors in orchard environments (Figure 15). Guo, Huipin et al. [123,124] integrated an improved A* algorithm with the dynamic window approach (DWA), alongside LiDAR and Gmapping for mapping, to realize autonomous navigation and obstacle avoidance in greenhouse tractors. Yang, Lili et al. [125] used quadratic programming and finite-state machines to study local path planning for dynamic and static obstacles on semi-structured farm roads. Zhou, Mingkuan et al. [126] combined the Hough matrix and RANSAC algorithm to extract starting paths for straw rotary tillage in rice fields.

Bio-inspired and swarm intelligence algorithms are also frequently applied in path planning. Chen, Hongtao et al. [127] developed a tractor obstacle-avoidance path-planning method using a genetic algorithm (GA) and Bézier curves. Liang, Chuandong et al. [128] and Li, Shengling et al. [129] explored multi-node path planning for electric tractors and comprehensive multi-plot planning in hilly areas, respectively, utilizing improved whale optimization algorithms (IWOA) and in combination with ant colony optimization (ACO). Liu, Li [130] optimized farm machinery operation paths with a particle swarm optimization algorithm. Xu, Shengli et al. [131] designed a pure vision autonomous navigation stack, integrating multi-task perception, coordinate transformation, and DWA, achieving full autonomy for tractors in peach orchards. For multi-camera obstacle detection, Zhou, Xinying et al. [132] proposed a YOLOv8 system, incorporating CBAM into its backbone and replacing BiFPN. Zhao, Xin et al. [133] introduced a collision-free path-planning method via the minimum snap algorithm.

**Figure 16:**
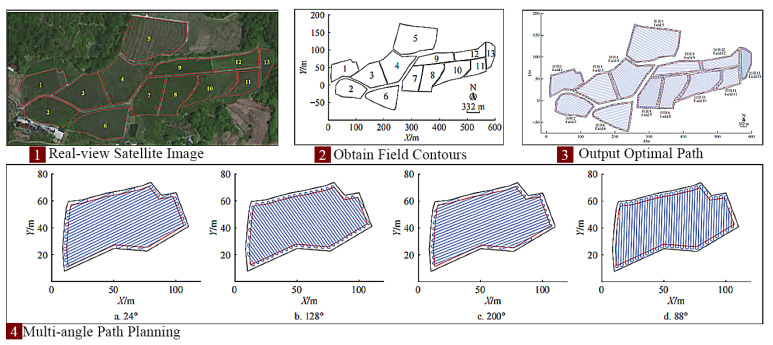
The full coverage operation path-planning technology for hilly–mountainous fields, integrating intelligent algorithms.

Integrating crop information is vital for path recognition and planning: Kachappilly, A et al. [134] used point cloud processing to assess and optimize paths based on crop damage risk. Soitina, Riikka et al. [135] presented a new coverage path-planning method for two cooperative autonomous tractors, ensuring collision-free operation through task decomposition and real-time scheduling. Fu, HT [136] introduced BiLG-D3QN, a deep reinforcement learning algorithm for segmented coverage path planning that balances load and energy. Liang, X [137] combined edge detection and the OTSU algorithm for path recognition in scenarios with broken rows and missing seedlings (Figure 16).

Based on the above research, the development directions for intelligent control technologies in soybean-harvesting equipment for China’s hilly–mountainous regions can be summarized as follows: (1) High-precision positioning and navigation systems: Integrating RTK-GNSS, IMU, LiDAR, and machine vision sensors (e.g., high-resolution RGB, multispectral cameras) [138] to achieve high-precision positioning and navigation. This enables harvesters to precisely align with rows in complex, irregular hilly plots, reducing missed areas and rework, thereby increasing operational efficiency [139]. (2) Intelligent path planning and obstacle avoidance: Utilizing high-precision digital elevation models, crop distribution maps, and real-time sensor data, AI algorithms will enable optimal operation path planning [140], automatically identifying and avoiding obstacles and hazardous areas (e.g., steep slopes, muddy zones, and ditches). In intercropping patterns, this includes precisely identifying soybean and corn strips to plan paths exclusively within soybean areas, preventing damage to corn plants [141,142]. (3) Human–machine collaboration and remote monitoring/diagnosis: This involves integrating remote monitoring, fault diagnosis, and early warning systems. Real-time machine data will be uploaded to a cloud platform via wireless communication, allowing operators to monitor machine status, operational parameters, and geographical information on tablets or in-cab displays. This facilitates remote analysis and troubleshooting, enhancing maintenance convenience and timeliness [143]. These directions follow a clear hierarchy, with high-precision positioning (1) forming the foundational prerequisite for effective path planning (2), and both enabling the rich data streams required for remote monitoring and collaboration (3).

Based on the extensive research reviewed in this study, the entire paradigm of intelligent navigation and path planning for harvesting in complex terrains is built upon the capabilities and integration of advanced sensor systems. This can be distilled into five fundamental pillars, where sensors are the primary subject:

(1) Proprioceptive sensors for self-awareness: The foundational sensory task for any autonomous machine is proprioception—knowing its own state. The research overwhelmingly identifies the fusion of RTK-GNSS, providing absolute centimeter-level positioning, with inertial measurement units (IMUs), delivering high-frequency attitude and orientation data, as the non-negotiable sensor suite for establishing this baseline of self-awareness.

(2) Exteroceptive sensors for 3D environmental modeling: To navigate safely, the machine must perceive the external world. LiDAR and stereo vision sensors are the primary exteroceptive tools responsible for this task. They actively build a real-time 3D model of the surrounding environment, which is fundamental for identifying and mapping terrain hazards, slopes, and both static and dynamic obstacles.

(3) Vision sensors for agronomic context perception: Beyond simple obstacle avoidance, intelligent harvesting requires an understanding of the agricultural context. Machine vision sensors, including RGB and multispectral cameras, act as the specialized sensory organs for this purpose. Powered by AI models, they perform the critical perception tasks of identifying crop rows for guidance, segmenting soybeans from corn for selective operations, and detecting field boundaries. Pilot systems utilizing such vision-based deep learning models have demonstrated over 95% accuracy in distinguishing target crops from weeds in real-time field conditions, providing essential performance evidence.

(4) Multi-sensor fusion as a core principle for robustness: A central theme is that no single-sensor modality is sufficient. The inherent weaknesses of one sensor (e.g., a camera’s vulnerability to lighting changes) are actively compensated for by the strengths of another (e.g., LiDAR’s consistent performance regardless of light). For instance, field trials have shown that fusing LiDAR and camera data can improve object detection reliability by over 20% in adverse lighting or dusty conditions compared to using a camera alone. Therefore, the principle of multi-sensor fusion is not an option but a necessity to create a single, holistic perception system that is far more reliable and robust than any individual sensor.

(5) Sensors as the indispensable input for action: Ultimately, the purpose of this entire sensory apparatus is to provide rich, actionable data to intelligent algorithms. The research pipeline is clear: sensors provide the “sight” and “feel,” which are processed into a world model. This model then serves as the critical input for path planning and control algorithms to translate perception into optimal, safe, and efficient physical action.

## 7. Precise Identification, Selective Harvesting, and Impurity Pre-Sorting for Soybeans in Intercropping Pattern

In a complex soybean–corn intercropping pattern, achieving precise soybean identification, selective harvesting, and effective impurity pre-treatment are critical technological challenges for improving harvest efficiency and quality.

### 7.1. Precise Soybean Crop Identification and Differentiation

Whether through advanced deep learning models like U-Net and MobileNet or traditional image processing techniques based on color and texture, the fundamental task is always the intelligent interpretation of visual data captured by the sensor.

**Figure 17:**
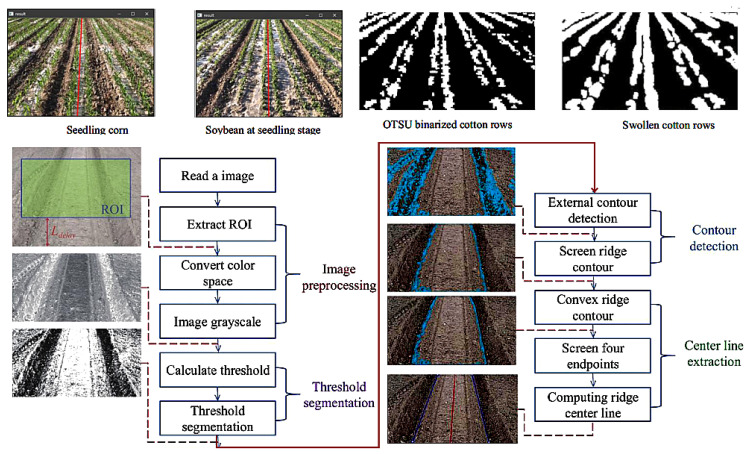
The crop edge extraction, row alignment, and field edge extraction technology.

Accurate crop identification is crucial for intelligent, automated harvesting, significantly boosting efficiency and final quality [144]. For precise crop differentiation and row alignment, Wang, Qian et al. [145] recognized wheat harvest boundary lines using semantic segmentation models. Lv, Runyi et al. [146] achieved real-time segmentation of crop-free ridges and fit navigation lines with the least squares method by replacing MobileNetV4. Kong, Xiangyu et al. [147] proposed an ENet network with an SE attention mechanism (SEU-ENet) for inter-row path recognition. Chen, Ziwen et al. [148] developed a lightweight Mobile-Unet, extracting traversable areas through semantic segmentation of targets like roads and tree trunks. Additionally, He, Sihan et al. [149] built an improved RCF (IRCF) deep learning model for identifying hilly field boundaries. Meanwhile, traditional machine vision methods still excel in specific scenarios: Liu, Wei et al. [150] recognized ridge centerlines using color features and grayscale reconstruction (Figure 17). Li, Xiangguang et al. [151] addressed corn seedling row recognition in cases of missing seedlings with HSV color space and morphological processing. Pan, Shengquan et al. [152] effectively distinguished harvested and unharvested wheat areas to extract operation boundary lines by constructing feature vectors of image entropy and orientation gradients.

The core meaning of these advancements lies in physically equipping the machine with a vision sensor—the ‘eye’ that is the absolute prerequisite for any subsequent automation and precision. Through the data provided by this sensor, harvesting machinery can, for the first time, truly understand its operating environment by distinguishing crops from non-crops, identifying boundaries, and aligning with rows. For this foundational perception layer, machine vision sensors, particularly high-resolution RGB cameras, are the most critical category, as they capture the essential color, texture, and morphological features required for semantic classification. This capability, originating directly from the sensor, is not an end in itself; rather, it serves as the foundational perception layer upon which all higher-level intelligent functions are built.

### 7.2. Multi-Sensor Fusion Applications Based on Deep Learning and Agronomic Perception

**Figure 18:**
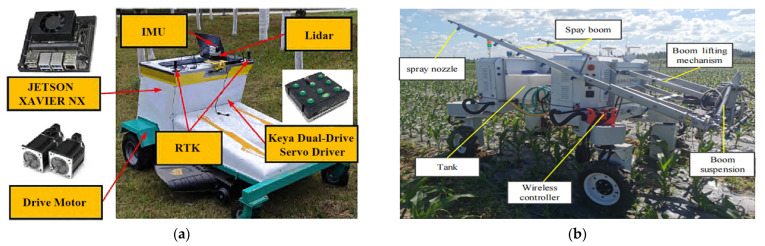
Research on multi-sensor harvesting equipment for hilly and mountainous areas. Design of a soybean header for complex terrain. (**a**) Multi-sensor fusion agricultural robot; (**b**) Multi-sensor fusion agricultural field management robot and its navigation control system.

Sensor fusion is widely employed in modern agricultural equipment design [153] (Figure 18). In macro-level vegetation and land cover classification, Stewart, Stephen B. et al. [154] effectively differentiated vegetation using Sentinel-1 (SAR) and Sentinel-2 (optical) satellite data. Lv, Jing et al. [155] fused four remote sensing data types, including radar, optical, and thermal infrared, for land feature identification. Similarly, Xiong, Sitian et al. [156] used optical and SAR data fusion with a Bayesian workflow to successfully identify and track farmland expansion. In agricultural robot environmental perception, multiple studies fuse on-board sensors for precise identification and navigation: He, Jing et al. [157] integrated LIDAR, AHRS, and RTK-GNSS for precise rice-row detection. The success of these deep learning models is critically dependent on large-scale, meticulously annotated datasets, often requiring thousands of images captured across diverse lighting conditions, growth stages, and geographical regions to ensure model robustness. Su, Zhengquan et al. [158], Hu, Xueting et al. [159], Ban, Chao et al. [160], and Liu, Yang et al. [161] achieved recognition of orchard tree trunks, corn plants, and inter-row navigation lines, along with robot localization, by fusing various sensor combinations including LIDAR, cameras, and IMU. However, the transferability of these models remains a significant challenge, as models trained in one region often require substantial re-training or fine-tuning to perform reliably in new environments with different crop varieties, soil types, and weed populations. Kang, Hanwen et al. [162] performed precise semantic segmentation of fruits in orchards using LIDAR–camera data fusion and deep learning. Additionally, Luo, Huanzhi et al. [163] used neural networks to fuse multi-sensor data for greenhouse environment state classification, while Tong, Rui et al. [164] built a fusion system to identify traffic targets like pedestrians and vehicles.

Based on the above research, a key direction for improving the research level of equipment in hilly–mountainous areas is as follows: Crop identification based on machine vision and deep learning. This is foundational for precise harvesting. By leveraging high-resolution RGB, multispectral, or hyperspectral imaging systems coupled with deep learning algorithms trained on vast field image datasets, it is possible to identify differences in morphological features, plant height, leaf color, texture, and growth stages of soybean and corn plants. This technology can even distinguish between lodged and healthy soybeans, enabling precise differentiation of soybeans, corn, and various weeds [165].

**Figure 19:**
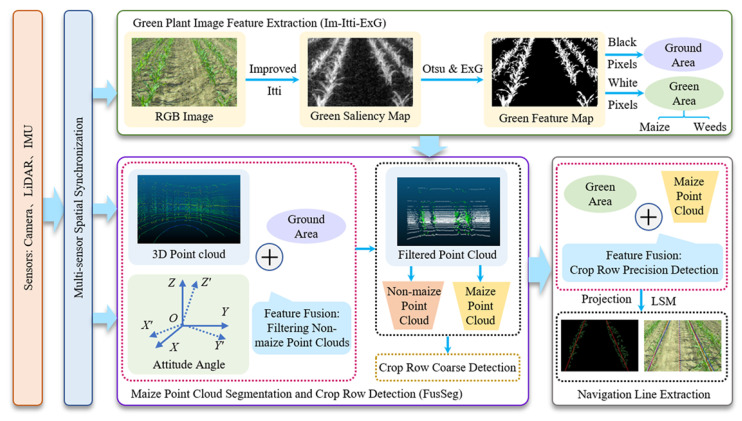
Multi-sensor agricultural robot technology based on camera-LiDAR-IMU fusion for real-time extraction of navigation lines between corn rows.

For agronomic feature perception, researchers precisely assess crop states by fusing various sensors: Long, Liu Yang et al. [161,166] integrated flowmeters, spectrometers, and LiDAR on combines for yield and environmental stress evaluation (Figure 19). Yuan et al. [167] and Schirrmann et al. [168] estimated wheat plant height and biomass using ground LiDAR combined with ultrasonic sensors or cameras. Chen et al. [169] and Xu et al. [170] comprehensively monitored corn nitrogen efficiency and multiple cotton phenotypic traits by fusing multispectral, thermal imaging, and LiDAR data. Rilling et al. [171] also employed a multi-sensor suite including hyperspectral, LiDAR, and thermal imaging for comprehensive crop detection. In agricultural robotics, multi-sensor fusion primarily addresses navigation and environmental perception: Ban et al. [160] and Cui et al. [172] fused LiDAR, cameras, and inertial measurement units (IMUs) for high-precision localization in GNSS-free environments, field navigation line extraction, and autonomous path tracking. Tan et al. and Li et al. [173,174] further researched fusing camera and LiDAR data for 3D semantic segmentation and object detection.

Based on the above research, another significant direction for advancing equipment research in hilly–mountainous areas is as follows: Multi-sensor fusion for recognition. By integrating crop height, 3D structure, and density information from LiDAR with visual data, it is possible to more accurately determine crop type and position. This significantly enhances recognition robustness and precision, especially in complex environments such as lodged soybeans, dense corn stalks, or insufficient light conditions.

In conclusion, the research across these diverse applications converges on a singular, powerful paradigm: the creation of a “logical super-sensor” through the fusion of multiple, disparate physical sensor modalities. Experimental evidence for this concept comes from autonomous navigation systems, where fusing LiDAR’s geometric data with a camera’s semantic data has been shown to improve crop-row detection accuracy by over 15% in variable lighting conditions, a task neither sensor could reliably perform alone. The underlying principle is that no single sensor can provide a complete picture. LiDAR, for example, offers precise geometric data (the “what shape and where”) but lacks semantic context. Conversely, a camera provides rich semantic and spectral information (the “what is it and what color”) but can struggle with accurate depth perception. The IMU adds the crucial context of the sensor platform’s own motion and orientation. By intelligently fusing these complementary data streams—the geometric, the semantic, and the inertial—the machine constructs a holistic and robust model of situational awareness. This fused perception is far more resilient to the failure or limitations of any single sensor (e.g., a camera blinded by sun glare), making multi-sensor fusion not just an optimization, but the essential enabling technology for achieving reliable and safe autonomous operation in the unstructured and unpredictable agricultural environment.

### 7.3. Deep Learning Applications for Advanced Soybean Perception

**Figure 20:**
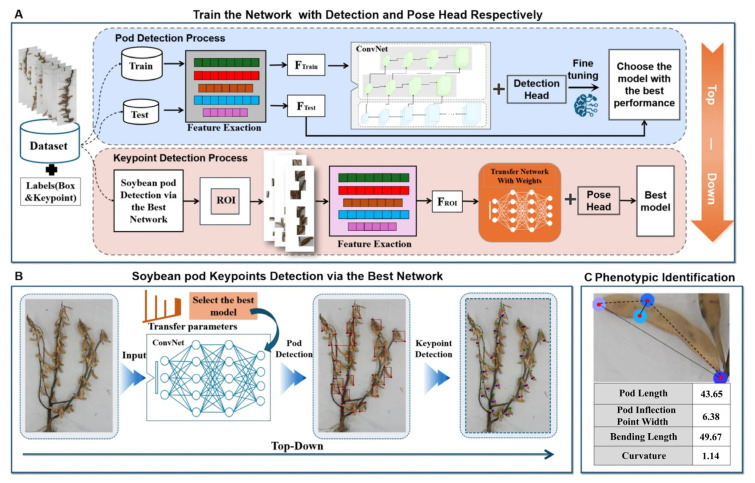
The experimental procedure of the Pod-pose method. (**A**) Training with separate object and keypoint detection heads. (**B**) Detecting keypoints using the trained model. (**C**) Identifying phenotypic traits based on detected keypoints.

Beyond navigation/localization, recent work shows that deep learning can extract granular, crop-specific traits that underpin intelligent harvesting. A major thrust targets organ-level perception—pod/seed detection, counting, and segmentation—with real-time instance segmentation (e.g., PodNet) and improved detector families (e.g., YOLOv8 variants), enabling precise pod localization directly from pre-harvest images [175,176]. These efforts are complemented by approaches that emphasize metric learning for finer pod discrimination (DLML-PC) and keypoint/pose estimation for fine-grained pod phenotyping (Pod-pose), as well as systems that operate across outdoor and indoor settings, improving generalization from field to lab [177]. Together, these advances support on-the-fly yield mapping and hotspot identification to guide header pathing and to inform real-time adjustments to threshing and cleaning intensity during harvest [178,179,180,181], as shown in Figure 20.

Building upward from organs to plants and fields, multimodal and time-series pipelines combine UAV imagery, point clouds, and dynamic modeling to deliver yield estimation and lodging discrimination at scale, while maintaining computational efficiency for practical deployment [182,183]. Region-level yield prediction has also benefited from multi-source remote sensing (e.g., fusing optical indices, texture, and topographic cues) optimized with deep learning, improving spatial coverage and stability in variable environments [184]. Although not always consumed directly as a real-time control input, these pre-harvest yield maps and lodging assessments enable better route planning and pre-setting of harvester parameters (e.g., baseline threshing speed and fan setpoints) matched to expected volume and moisture conditions, which can reduce losses during peak operations.

At the canopy/leaf and seed scales, deep learning is advancing disease recognition and varietal identification. Improved YOLOv8 models now handle disease detection in complex field scenes, extending earlier transfer-learning pipelines that established robust baselines for in situ diagnosis [185,186]. For variety identification, hyperspectral imaging combined with deep learning (including attention mechanisms and transfer learning) discriminates soybean seed varieties with high accuracy, creating a path to sensor-informed machine presets (e.g., accounting for differences in pod shatter resistance, seed size, and moisture behavior across cultivars) [187,188]. In a harvesting context, disease and variety cues can support selective treatment, segregated grain handling, and variety-aware control policies that fine-tune reel, header, and cleaning settings to minimize damage and impurity rates [189].

Implication for intelligent harvesting: Collectively, these DL-driven perception capabilities provide the crop-specific observables needed to move from fixed settings to context-aware control—linking pod/plant signals to row—following and header targeting, and linking yield/lodging/disease/variety signals to adaptive threshing and cleaning strategies that pursue low damage and high efficiency throughout the harvest window.

### 7.4. Research on Precise Cutting and Selective Harvesting of Soybeans

In agricultural harvesting technology and equipment research, scholars have approached challenges in complex agronomic systems from multiple dimensions. For core header precision control and structural innovation, Zhou, Weiwei [96] and Nie, Junshan [190] designed profiling mechanisms (based on probe wheels, sensors, and hydraulic systems). Combined with multi-body dynamics simulation and experimental validation, this achieved precise automatic control of header height, addressing missed cuts and soil-scooping issues during low-lying crop harvest. Simultaneously, to adapt to specific planting patterns, Cao, Zhipeng [191] and Luo, Huizhong [192] developed specialized headers for soybean–corn intercropping through structural design and finite element analysis to enable selective harvesting. Hou Jie [193] designed a variable-structure universal header adaptable to various crops. For refined control of harvesting components, Leng, Yuancai [95] and Jin, Chengqian [68] used PLC control or co-simulation to design reel control systems that automatically adjust speed based on crop conditions, reducing feeding loss. Regarding harvest process optimization, Cortez, Jorge, W. [194], de Menezes, Patricia, C. [195], and NI, Youliang [196] compared the impact of different header types and operational speeds on total losses, systematically analyzing the mechanisms of soybean breakage during various stages of mechanical harvesting.

Evidently, the optimization directions for precise cutting and selective harvesting of soybeans are fundamentally sensor-driven control problems. They primarily involve three points:

(1) Intelligent header and dynamic cutting control: This is a classic closed-loop system where real-time data from a machine vision or LiDAR sensor system, which performs the crop identification, directly drives the control commands. While extensive field trials of such integrated intelligent headers specifically in Southwest China are still limited, prototypes tested in comparable uneven terrains in other regions have demonstrated the capability to precisely follow ground contours, validating the technical feasibility of the concept. Based on this continuous stream of sensor data, electro-hydraulic proportional valves or servo motors precisely control the header’s lateral position and cutter penetration depth. This sensor-guided actuation is what enables the harvester to “intelligently” and precisely cut only soybean plants within intercropped strips, avoiding damage to adjacent corn.

(2) Optimization of row and plant separation mechanisms: Rather than being purely passive, advanced dividers are active systems dynamically informed by proximity sensors or localized machine vision. These sensors detect the precise location of corn stalks relative to soybean plants, allowing for real-time adjustments to physical dividers or airflow intensity. This ensures that soybean plants are guided smoothly into the harvesting area while corn plants are deflected, achieving efficient collection without corn damage based on direct sensory feedback.

(3) Modular and independent header systems: The concept of modularity extends to the sensory system, creating a distributed sensing and control architecture. Each independent mini-header is equipped with its own dedicated set of sensors—typically a terrain-profiling sensor for height and a vision sensor for row tracking. This allows each module to independently perceive its local environment and adjust its height, angle, and lateral position, enabling highly adaptive and precise harvesting of different rows or crop strips simultaneously.

## 8. Low-Damage, High-Efficiency Soybean Threshing and Optimized Cleaning Performance for Hilly–Mountainous Slopes

**Figure 21:**
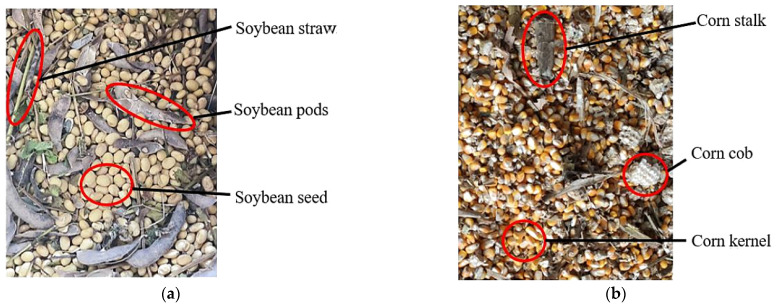
Analysis of incomplete threshing during soybean harvesting with existing equipment in hilly and mountainous areas. (**a**) Incomplete threshing residues after soybean harvest; (**b**) Incomplete threshing residues after corn harvest.

Post-harvest grain quality (breakage, impurity content) directly impacts soybean commercial value and storage. Optimizing threshing and cleaning technologies is crucial for enhancing harvest benefits and reducing economic losses [197]. Researchers have used various methods to optimize threshing devices, aiming to reduce breakage and losses: Kang Jiaxin [198,199,200], Tan Yunfeng [201], and Jin Chenggian et al. [202] designed novel threshing mechanisms (e.g., differential drums, flexible curved teeth, and adjustable concaves) through experimental studies and software simulations, optimizing their structure and operating parameters for improved performance and material adaptability. Among these innovations, the development of flexible threshing elements holds the most significant potential for soybean harvesting specifically, as their compliant nature can drastically reduce the high-impact forces that lead to grain breakage and damage, a primary concern for this crop. Hussain, S. [203], Xu Yuanyu [204], and NI, Youliang et al. [196] systematically investigated the influence of operational parameters—like drum speed, forward speed, and feed rate—on threshed material distribution, loss rate, and breakage patterns via field experiments and factor analysis, identifying optimal combinations. Additionally, Chen, Yuxuan [205,206], Gun-Ho, Lee [207], Tan Yunfeng [208], and Ho, Lee Gun [209] et al. employed finite element/discrete element simulations and physical tests to microscopically analyze stress distribution, damage mechanisms, force characteristics, and the impact of moisture content on grain strength during soybean collisions, providing a theoretical basis for damage-reducing threshing component design (Figure 21).

**Figure 22:**
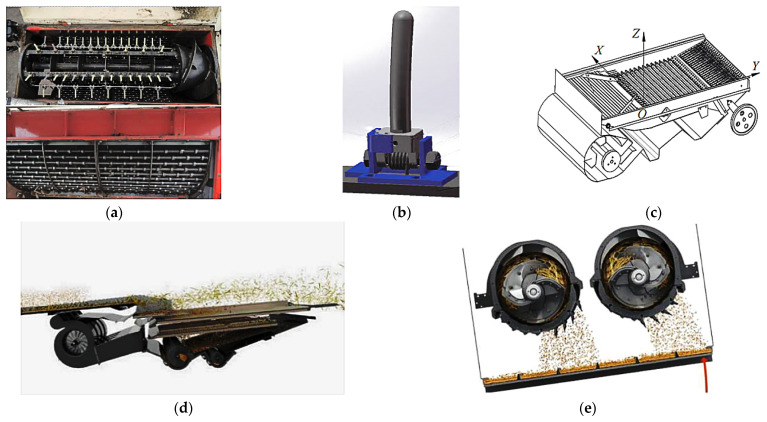
Optimized development directions for threshing and separation technologies in soybean harvesting. (**a**) Axial-flow threshing drum; (**b**) flexible threshing element; (**c**) crop screening structure; (**d**) airflow-assisted threshing; (**e**) intelligent optimization and adaptive adjustment of threshing parameters (including 4D cleaning technology).

To enhance threshing efficiency and quality, researchers have explored intelligent control, structural optimization, and fault diagnosis from multiple perspectives [210]. In intelligent control, Guo, Dafang et al. [211,212] utilized digital twin (DT) technology to build online optimization methods and system architectures for reducing breakage rates and improving efficiency. Li, Xiaoyu et al. [213] designed a low-damage corn-threshing automatic control system using an improved particle swarm optimization and cuckoo search algorithm. Li, Yang et al. [214] proposed a multi-parameter collaborative control method for threshing and cleaning based on model predictive control (MPC). For structural optimization (Figure 22), Wu, Luofa et al. [215] designed an adjustable straw guide plate using response surface methodology to promote timely grain–straw separation and reduce subsequent losses. Zhang, Dongming et al. [216] optimized the drum structure of a millet threshing device for axial-flow threshing and separation units using the discrete element method (DEM). To ensure threshing stability, Wang, Suzhen et al. [217] analyzed vibration signals with Hilbert–Huang transform for high-precision prediction of threshing drum blockages. Wang, Jinwu et al. [218] dynamically optimized the core component of the longitudinal axial-flow threshing drum to enhance separation performance. To suit special operating environments like hilly–mountainous areas and breeding fields [219], Luo Hongbo and Ni Youlian et al. designed small segmented and specialized low-loss combine harvesters, respectively. Chen, Man [220] and Jin Chengqi [221] developed harvesting equipment with online intelligent monitoring systems for real-time tracking of grain breakage and impurities. Wang, Ruochen [222] proposed a structural design solution based on a three-layer frame with a hydraulically interconnected omnidirectional leveling system, ensuring platform stability by maintaining a fixed center point during leveling.

Based on the above research, the optimization of threshing and separation/cleaning technologies for soybean harvesting primarily focuses on two key areas: low-damage threshing technology optimization and high-efficiency separation and cleaning technology optimization Figure 39 in [223].

Specifically, low-damage threshing technology optimization includes the improved design of axial-flow threshing drums, flexible threshing elements, airflow-assisted threshing, and intelligent regulation of threshing parameters [224]. Meanwhile, high-efficiency separation and cleaning technology optimization encompasses features such as automatic slope compensation cleaning systems, multi-stage cleaning with air-screen optimization [225], intelligent adaptive adjustment of cleaning parameters [226], and advanced designs for residue treatment and grain recovery [227]. Among these, low-damage threshing technology represents the more urgent priority for real-world adoption, as grain breakage during this initial stage is irreversible and causes a direct, significant loss of market value.

Intelligent control strategies—e.g., Digital Twins and MPC—depend on continuous, high-quality sensor data. Vibration and torque sensors support early detection of overloads and impending blockages; machine vision and acoustic sensors provide real-time indicators of grain breakage and impurity levels for closed-loop adjustment. Mass-flow and moisture sensors characterize the incoming crop stream, enabling continuous adaptation of threshing intensity and cleaning settings to field variability. Collectively, these sensors provide the observability required to transform threshing and cleaning from fixed-setting mechanisms into adaptive systems that pursue low damage and high efficiency.

## 9. Conclusions

Fulfilling its objectives, this review has identified critical sensor-driven technological frontiers, analyzed the systemic mismatch of current machinery from a perception-centric standpoint, and laid out a technological roadmap for future intelligent systems. This review has critically assessed the state of soybean harvest mechanization in Southwest China’s hilly–mountainous intercropping systems, revealing that the persistent bottleneck is not merely a collection of isolated technical issues, but a symptom of a fundamental disconnect between current agricultural machinery engineering paradigms and the complex, heterogeneous nature of these agro-ecosystems. Our principal conclusions are as follows: (1) Systemic mismatch, not just component failure: The core challenge transcends the limitations of individual components. It is a systemic problem, where the machine, the plant, and the terrain form a tightly coupled, complex system. Current harvesters, often scaled-down versions of designs for homogenous plains, fail because they lack the requisite adaptability and situational awareness to operate within this system’s constraints, such as asynchronous crop maturity, irregular plot boundaries, and variable slopes. (2) The imperative for “Sensing-Thinking-Acting” machinery: The analysis of crop damage patterns and existing technological attempts underscores that true progress lies beyond simple mechanical improvements. The future demands the deep integration of advanced sensing (to perceive the complex environment), artificial intelligence (to make real-time decisions), and mechatronic actuation (to execute tasks with precision). This signifies a necessary evolution from automated machines to genuinely autonomous, context-aware robotic systems. (3) A new design philosophy: From “One-Size-Fits-All” to “Specialized and Adaptable”: This review consolidates the evidence that a new design philosophy is essential. However, this paradigm shift is currently limited by significant barriers, including the high initial R&D and acquisition costs of specialized machinery, the complexity of system integration and maintenance, and the lack of a supporting service infrastructure in rural regions. Instead of pursuing a universal harvester, the focus must shift to developing a portfolio of compact, modular, and intelligent machines specifically engineered for the challenges of intercropping and rugged terrain. This approach prioritizes maneuverability, precision, and efficiency over raw power and scale. In essence, overcoming the soybean-harvesting bottleneck requires a paradigm shift in the engineering of agricultural machines. The focus must evolve from designing machines that impose uniformity on the environment to creating intelligent systems that can adapt and thrive within their inherent complexity. Overall, this review provides a sensor-based framework for mechanizing maize–soybean intercropping in hilly–mountainous regions, linking field constraints to sensing modalities, perception algorithms, and control actions to guide deployable, low-loss harvesting solutions.

## 10. Prospects

Addressing the mechanization bottleneck in soybean harvesting under challenging agro-terrains requires a coordinated, interdisciplinary effort. Based on the challenges and technology gaps identified in this review, future work should prioritize the following:

(1) Integrated system modeling and design optimization. Progress depends on moving beyond component-level tuning to holistic, system-level design. A key direction is building high-fidelity simulation platforms that couple harvester dynamics with crop models (plant architecture and mechanical properties) and operational environments. Such integrated models provide virtual testbeds to optimize machine parameters, evaluate concepts, and shorten costly prototype-field test cycles.

(2) Compact, modular, and cooperative machinery. Given small, fragmented, and irregular plots, relying on a single large harvester is often impractical. Research should focus on compact, modular units with enhanced maneuverability on slopes and in tight spaces, operating individually or in cooperative multi-robot formations. Priorities include robust communication, distributed task allocation, and safe, efficient multi-machine navigation in complex fields.

(3) Enhanced environmental perception and intelligent control. Reliability and efficiency hinge on the ability to perceive and adapt. Fusing LiDAR, multispectral/RGB cameras, and IMUs should yield a robust, real-time field representation. This perception stack should drive adaptive control, e.g., dynamic header-height adjustment from terrain profiling, threshing intensity modulation based on volume/moisture, and precise row-following for selective harvesting in intercropped patterns.

(4) Agri-machinery integration and co-design. Sustainable solutions will come from tighter collaboration between agricultural engineers and agronomists. A two-way feedback loop is essential: machine performance data should inform intercropping design (row spacing and plant density) to improve mechanization, while machinery design must respect biological constraints. Joint efforts can also support targeted breeding (including genome editing) for “mechanization-friendly” varieties with higher lowest-pod height, improved lodging resistance, and more uniform maturation.

Pursuing these avenues systematically can enable a new generation of context-appropriate, sensor-driven machinery for soybean harvesting in hilly–mountainous, intercropped systems—advancing both technological capability and field practicality.

## Data Availability

The data and the related conclusions presented in this article were all derived from the Web of Science database and “CNKI” (China National Knowledge Infrastructure).
